# MicroRNAs participate in the regulation of apoptosis and oxidative stress-related gene expression in rabbits infected with *Lagovirus europaeus* GI.1 and GI.2 genotypes

**DOI:** 10.3389/fmicb.2024.1349535

**Published:** 2024-03-07

**Authors:** Ewa Ostrycharz, Andrzej Fitzner, Andrzej Kęsy, Aldona Siennicka, Beata Hukowska-Szematowicz

**Affiliations:** ^1^Institute of Biology, University of Szczecin, Szczecin, Poland; ^2^Doctoral School, University of Szczecin, Szczecin, Poland; ^3^Molecular Biology and Biotechnology Center, University of Szczecin, Szczecin, Poland; ^4^Department of Foot and Mouth Disease, National Veterinary Research Institute-State Research Institute, Zduńska Wola, Poland; ^5^National Reference Laboratory for Rabbit Hemorrhagic Disease (RHD), Zduńska Wola, Poland; ^6^Department of Laboratory Diagnostics, Pomeranian Medical University, Szczecin, Poland

**Keywords:** *Lagovirus europaeus*, RHDV, rabbit hemorrhagic disease (RHD), miRs, apoptosis, oxidative stress, rabbits, biomarker

## Abstract

MicroRNAs (miRs) are a group of small, 17–25 nucleotide, non-coding RNA that regulate gene expression at the post-transcriptional level. To date, little is known about the molecular signatures of regulatory interactions between miRs and apoptosis and oxidative stress in viral diseases. *Lagovirus europaeus* is a virus that causes severe disease in rabbits (*Oryctolagus cuniculus*) called Rabbit Hemorrhagic Disease (RHD) and belongs to the *Caliciviridae* family, *Lagovirus* genus. Within *Lagovirus europaeus* associated with RHD, two genotypes (GI.1 and GI.2) have been distinguished, and the GI.1 genotype includes four variants (GI.1a, GI.1b, GI.1c, and GI.1d). The study aimed to assess the expression of miRs and their target genes involved in apoptosis and oxidative stress, as well as their potential impact on the pathways during *Lagovirus europaeus*—two genotypes (GI.1 and GI.2) infection of different virulences in four tissues (liver, lung, kidneys, and spleen). The expression of miRs and target genes related to apoptosis and oxidative stress was determined using quantitative real-time PCR (qPCR). In this study, we evaluated the expression of miR-21 (*PTEN*, *PDCD4*), miR-16b (*Bcl-2*, *CXCL10*), miR-34a (*p53*, *SIRT1*), and miRs—related to oxidative stress—miR-122 (*Bach1*) and miR-132 (*Nfr-2*). We also examined the biomarkers of both processes (*Bax*, *Bax/Bcl-2* ratio, *Caspase-3*, *PARP*) and *HO-I* as biomarkers of oxidative stress. Our report is the first to present the regulatory effects of miRs on apoptosis and oxidative stress genes in rabbit infection with *Lagovirus europaeus*—two genotypes (GI.1 and GI.2) in four tissues (liver, lungs, kidneys, and spleen). The regulatory effect of miRs indicates that, on the one hand, miRs can intensify apoptosis (miR-16b, miR-34a) in the examined organs in response to a viral stimulus and, on the other hand, inhibit (miR-21), which in both cases may be a determinant of the pathogenesis of RHD and tissue damage. Biomarkers of the *Bax* and *Bax*/*Bcl-2* ratio promote more intense apoptosis after infection with the *Lagovirus europaeus* GI.2 genotype. Our findings demonstrate that miR-122 and miR-132 regulate oxidative stress in the pathogenesis of RHD, which is associated with tissue damage. The *HO-1* biomarker in the course of rabbit hemorrhagic disease indicates oxidative tissue damage. Our findings show that miR-21, miR-16b, and miR-34a regulate three apoptosis pathways. Meanwhile, miR-122 and miR-132 are involved in two oxidative stress pathways.

## 1 Introduction

MicroRNA molecules (miRs) are single-stranded, non-coding ribonucleic acid sequences of 17–25 nucleotides in length, playing an essential role in the post-transcriptional regulation of gene expression. They are created from double-stranded precursors produced by RNA polymerase II (Bartel, 2004).

Our recent studies indicate the role of miRs as micro-players of great importance in viral infections in humans and animals (Hukowska-Szematowicz et al., 2020; Ostrycharz and Hukowska-Szematowicz, 2022; Hukowska-Szematowicz et al., 2023). miRs can regulate gene expression related to apoptosis (Su et al., 2015) and oxidative stress (Banerjee et al., 2017; Fioravanti et al., 2022) during diseases, including viral ones (Hukowska-Szematowicz et al., 2020; Fioravanti et al., 2022; Ostrycharz and Hukowska-Szematowicz, 2022), and thus affect the activity status of signaling pathways (Chen, 2015; Sadri Nahand et al., 2021).

Apoptosis and oxidative balance maintain cell homeostasis and play an essential role in viral infections (Thomson, 2001; Santus et al., 2022). Many cells undergo apoptosis in response to viral infection, reducing the release of progeny virus. During viral infection, the relative expression of virus-related genes and the activation of innate antiviral response systems lead to an increase in reactive oxygen species (ROS), reactive nitrogen species (RNS), and toxic of by products energy metabolism. The imbalance between ROS and antioxidants in the body leads to oxidative stress, cell death, and tissue/organ damage in the host. ROS and the resulting changes in cellular redox status become one of the inducing factors for cell apoptosis (Valyi-Nagy and Dermody, 2005). Tissue damage related to apoptosis and oxidative stress is an essential element in the pathogenesis of infection with coronaviruses (Gain et al., 2022), herpesviruses (Sun et al., 2023), rhabdoviruses (Verma et al., 2018), and paramyxoviruses (Yang et al., 2023). So far, little is known about the molecular signatures of regulatory interactions between specific miRs and apoptosis and oxidative stress in diseases (Su et al., 2015; Banerjee et al., 2017; Klieser et al., 2019; Sadri Nahand et al., 2021; Fioravanti et al., 2022; Abolfathi et al., 2023). No studies are addressing this scientific problem in viral diseases. Understanding these interactions has diagnostic (searching for potential disease biomarkers) and therapeutic (modulating miR-dependent pathways) potential in the course of acute liver failure (ALF) and organ dysfunction in multi-organ failure (MOF) of viral etiology, which we encounter during *Lagovirus europaeus* infection (Tunon et al., 2003; Esteves et al., 2018; Asim et al., 2020).

*Lagovirus europaeus* (*L. europaeus*) genotypes GI.1 and GI.2 are etiological factors for Rabbit Hemorrhagic Disease (RHD), belonging to the *Caliciviridae* family, *Lagovirus* genus (Le Pendu et al., 2017). *Lagovirus europaeus* is a single-stranded RNA virus (Le Pendu et al., 2017). The *L. europaeus* GI.1 genotype includes four variants (GI.1a/GI.1b, GI.1c, and GI.1d), with the GI.1a variant being highly pathogenic and most often causing an acute form of the disease, with mortality rates of 90%–100%. However, the *L. europaeus* GI.2 genotype evokes per-acute, acute, subacute chronic, and subclinical forms of the disease with variable mortality of 50%–80%, depending on the strain (Rocchi and Dagleish, 2018).

Although the first RHD epidemic occurred 39 years ago, its pathogenesis mechanisms are still not fully understood (Liu et al., 1984). During RHD, many pathological changes occur in the rabbit’s organs, especially the liver (the site of virus replication), lung, spleen, and kidney (Abrantes et al., 2012). The highest titer of *L. europaeus* is found in the liver, lung, spleen, kidneys, trachea, and bone marrow, which are the most frequently assessed organs in the diagnostic process (Abrantes et al., 2012; Rocchi and Dagleish, 2018). The main changes in RHD are acute liver, spleen, lung, and kidney inflammation. Pathological changes include enlargement of the liver, spleen (splenomegaly), and kidneys, as well as hyperemia and pulmonary edema (results in severe failure manifesting as shortness of breath, worsening by death; Abrantes et al., 2012; Rocchi and Dagleish, 2018). In these organs, inflammatory foci rich in neutrophils and T and B lymphocytes are observed, as well as an increase in the expression of the inflammatory biomarker—miR-155-5p—in the liver, lungs, and kidneys and a decrease in the spleen (Hukowska-Szematowicz et al., 2023). It is known that systemic hemorrhagic diathesis and intravascular coagulation syndrome (DIC) can lead to death in rabbits (Trzeciak-Ryczek et al., 2015). Innate and adaptive immunity also play an essential role in the pathogenesis of RHD (Hukowska-Szematowicz, 2013), including peripheral blood leukocytes (Trzeciak-Ryczek et al., 2016, 2017; Semerjyan et al., 2019). After *L. europaeus* infection, the level of pro-inflammatory and anti-inflammatory cytokines increases in the serum, liver, and spleen (Teixeira et al., 2012; Trzeciak-Ryczek et al., 2016; Ostrycharz et al., 2021; O'Toole et al., 2022).

It has also been shown that apoptosis (Alonso et al., 1998; Jung et al., 2000; San-Miguel et al., 2006; Ni et al., 2009; Garcia-Lastra et al., 2010; Marques et al., 2010; Tunon et al., 2011; Chen et al., 2018), necrosis (Park et al., 1995), and oxidative stress (San-Miguel et al., 2006; Hu et al., 2020) are elements in the pathogenesis of RHD. The progression of the disease correlates with increased apoptosis not only of liver cells (Alonso et al., 1998; Jung et al., 2000; Garcia-Lastra et al., 2010; Tunon et al., 2011; Vallejo et al., 2014; Chen et al., 2018; Bebnowska et al., 2024) but also of lung, kidney, heart, and spleen cells (Alonso et al., 1998; San-Miguel et al., 2006; Ni et al., 2009; Bebnowska et al., 2023), as well as necrosis (Park et al., 1995). Further studies showed apoptosis of T and B lymphocytes in the liver, spleen, and granulocytes and lymphocytes in peripheral blood (Marques et al., 2010; Niedzwiedzka-Rystwej and Deptula, 2012; Hukowska-Szematowicz, 2013; Niedzwiedzka-Rystwej et al., 2013a,b). Hukowska-Szematowicz et al. (2020) showed that after infection of rabbits with the *L. europaeus* GI.1 genotype, the expression of proapoptotic miR-16-5p increased in the liver. The same studies did not show changes in the liver’s expression of miR-122-5p (potentially involved in oxidative stress) but were detected in the serum. In the same year, Hu et al. (2020) showed that oxidative stress plays a crucial role in liver damage, especially in early RHDV infection (18 h p.i.). The nuclear translocation of Keap1-NF-kB is crucial for suppressing the NFR-2-ARE pathway in hepatocytes. Upregulation of Nrf-2 protein levels in liver cell nuclei by tert-butylhydroquinone (tBHQ) delayed the death of rabbits with RHDV infection. Earlier in 2006, San-Miguel et al. (2006) showed that oxidative stress is the main apoptosis pathway in RHD.

The above findings indicate that apoptosis and oxidative stress play an essential role in the pathogenesis of RHD. However, so far [apart from the research of Hukowska-Szematowicz et al. (2020) and Ostrycharz and Hukowska-Szematowicz (2022)], nothing is known about the molecular signatures of regulatory interactions between miRs and apoptosis and oxidative stress in *L. europaeus* infection/pathogenesis of RHD.

Therefore, we undertook research aimed at assessing the expression of miRs and their target genes related to apoptosis and oxidative stress and assessing their potential impact on the pathways in infection with *L. europaeus*—two genotypes (GI.1 and GI.2) with different virulence in four tissues (liver, lungs, kidneys, and spleen). Based on *in silico* analysis and our previous literature data (Hukowska-Szematowicz et al., 2020; Ostrycharz and Hukowska-Szematowicz, 2022), we selected miRs/target genes in the apoptosis and oxidative stress processes. For apoptosis: miR-21 (target genes—phosphatase and tensin homolog [*PTEN*] and programmed cell death factor 4 [*PDCD4*]), miR-16b (antiapoptotic B cell lymphoma 2 [*Bcl-2*] and C-X-C motif chemokine ligand 10 [*CXCL10*]), miR-34a (p53 protein gene (*p53*), and silent information regulator 1 [*SIRT1*]). For oxidative stress: miR-122 (BTB domain and CNC homolog 1 [*Bach1*]); miR-132 (nuclear factor erythroid 2-related factor 2 [*Nfr-2*]). We also examined biomarkers of both processes in rabbits infected with *L. europaeus* GI.1 and GI.2 genotypes: *Bax*, *Bax/Bcl-2* ratio, poly (ADP-ribose) polymerase (*PARP*), *Caspase-3*, and heme oxygenase-1 (*HO-I*) as a biomarker of oxidative stress.

## 2 Materials and methods

### 2.1 Ethical statements

The experiment was conducted in the experimental animal facility of the Pomeranian Medical University (PUM) in Szczecin. The experiment was approved by the Local Ethical Committee for Animal Experiments in Poznań, Poland (no. 51/2022). Rabbits were maintained according to European Union and national guidelines for animal experimentation.

### 2.2 Viruses

Two viruses were used to provoke infection in rabbits—*L. europaeus* (Fitzner et al., 2021). *Lagovirus europaeus* genotype GI.1, variant GI.1a, was named BBI (Poland, 2017; GenBank accession no. MG602005) and *L. europaeus* genotype GI.2 was named PIN (Poland, 2018; GenBank accession no. MN853660). Both viruses were titer-determined by the hemagglutination (HA) assay. The infectious titer of the *L. europaeus* GI.1 genotype inoculum (1 mL) was determined to be 0.5 u/mL (1 HA unit corresponds to 10^4^ particles per ml). The infectious titer of the *L. europaeus* GI.2 genotype inoculum (1 mL) was determined to be 2.048 u/mL (1 HA unit corresponds to 10^4^ particles per ml). The viruses have been prepared at the National Reference Laboratory for Rabbit Hemorrhagic Disease (RHD) and the Department of Foot and Mouth Disease, the National Veterinary Research Institute—State Research Institute in Zduńska Wola, Poland.

### 2.3 Experimental model

The study involved 30 European rabbits, *Oryctolagus cuniculus*—Crl:KBL (NZW)/052, both sexes (50:50 ratio), 6-month-old, body weights 4.0–4.5 kg, purchased from AnimaLab Limited Liability Company (branch in Poland, Poznań), and a 3-week adaptation period occurred after the animals were delivered to the university’s experimental facility. The animals had constant access to entertainment in their living environment, such as wooden chews and tunnels filled with hay. The recommended national guideline standards developed per the European Union Directive during the experiment ensured appropriate zoo-technical conditions. Autonomous air conditioning provided a temperature of 22°C (±1°C), 50%–60% humidity, and 15–20 air changes per hour. The animals stayed in rooms with artificial lighting, automatically controlled (12 h of light/12 h of darkness), and red night lighting. Both water and food were available to the animals *ad libitum*. Animals were randomly divided into three groups. The control group (*n* = 10) was injected intramuscularly with PBS (phosphate-buffered saline; 1 mL) as a placebo. Rabbits in the infection groups were injected intramuscularly with 1 mL virus. Group 2 received the *L. europaeus* GI.1 genotype (named BBI strain, Poland 2017). Group 3 received the *L. europaeus* GI.2 genotype (PIN strain, Poland 2018). Administration of the antigen in the infected rabbit group and PBS in the control group was marked as the beginning of the experiment. After this time, the animals’ health was monitored by measuring body temperature and registering clinical signs. After the infected animals showed severe symptoms of the disease, confirmed by a doctor, they were subjected to a euthanasia procedure as a result of intravenous administration of an anesthetic agent (ketamine 35–50 mg/kg; xylazine 5–10 mg/kg), followed by administration of the drug causing cardiac arrest, sodium pentobarbital (at 240 mg/kg).

### 2.4 Tissue sample collection

Tissue samples from the liver, lungs, spleen, and kidneys were obtained from the infected animals (20 rabbits) post-mortem or euthanasia, clinically defined. The organs were taken from the healthy control animals (10 rabbits) after euthanasia (as described above). Each tissue sample was washed in cold PBS and immediately placed in liquid nitrogen. Tissue samples were stored at −80°C until mRNA/miRs extraction.

### 2.5 Selection and *in silico* prediction of miRs target genes involved in the apoptosis and oxidative stress in *Oryctolagus cuniculus*

The process included several stages. In the first stage, miRs involved in apoptosis and oxidative stress processes were selected (Hukowska-Szematowicz et al., 2020; Ostrycharz and Hukowska-Szematowicz, 2022). At this stage, the miRTarBase database (Chou et al., 2018) was used to select miRs using various search strategies (by miRs, by target gene, by pathway/process, by validated methods, by disease). The criterion for selecting miRs was validation methods: strong evidence and our previous literature data (Hukowska-Szematowicz et al., 2020; Ostrycharz and Hukowska-Szematowicz, 2022). *Homo sapiens* miRs with a described role in the apoptosis process (miR-21, miR-16b, miR-34a) and oxidative stress (miR-122, miR-132) were selected (Chou et al., 2018; Hukowska-Szematowicz et al., 2020; Ostrycharz and Hukowska-Szematowicz, 2022). In the second stage, the miRTarBase database (Chou et al., 2018) and miRDB database (Chen and Wang, 2020) were used to select target genes (for chosen miRs) previously validated by RT-qPCR, Western blot, or a reporter assay in other species (validation methods: strong evidence). Next, the set of genes was used to conduct a gene ontology (GO) analysis via a GO enrichment analysis powered by protein annotation through evolutionary relationship (PANTHER; Ashburner et al., 2000). The analysis included: analysis type: PANTHER overrepresentation test; reference list: all *Homo sapiens* genes in the database; annotation data set: GO biological process complete; test type: Fisher’s exact; and correction: calculate false discovery rate (FDR). From all the processes with FDR *p <* 0.05, those correlated with apoptosis, oxidative stress, liver diseases, and multi-organ dysfunction in humans and animals were used for further steps. The 3′-UTR sequences of the *Oryctolagus cuniculus* genes involved in the selected processes were assessed to determine if they featured binding sites for miR-21-5p, miR-16-5p, miR-34a-5p, miR-122-5p, and miR-132-5p using the TargetScan database (Agarwal et al., 2015). In the third stage, to verify the importance of miR-21-5p, miR-16-5p, miR-34a-5p, miR-122-5p, and miR-132-5p in *L. europaeus* infection, an *in silico* analysis of putative target genes was conducted. Due to the inability to use one database to demonstrate the miR–mRNA interactions in *Oryctolagus cuniculus,* the following approach was selected: (i) mature sequences of these miRs in *Oryctolagus cuniculus* and *Homo sapiens* were compared, and no differences were found. We decided to use miRTarBase (Chou et al., 2018), which lists genes with validated miR–mRNA interactions by RT-qPCR or luciferase assays in *Homo sapiens.* Ultimately, five lists containing 53 for miR-21-5p, 36 for miR-16b-5p, 80 for miR-34a-5p, 18 target genes for miR-122-5p, and 18 for miR-132-5p were created; (ii) there was an attempt to determine the processes related to RHD that miR-21-5p, miR-16-5p, miR-34a-5p, miR-122-5p, and miR-132-5p might regulate. For this purpose, a GO analysis was conducted on the putative target genes for every miR separately. Thus, 52 processes for miR-21-5p, 1,152 processes for miR-16b-5p, 53 processes for miR-34a-5p, 80 processes for miR-122-5p, and 50 processes for miR-132-5p were identified. From these three groups, processes that correlated with RHD pathogenesis, ALF, and MOF were chosen for further analysis: 12 for miR-21-5p, 14 for miR-16b-5p, 14 for miR-34-5p, 7 for miR-122-5p, and 8 for miR-132-5p. At this step, all analyses were performed based on miR–mRNA interactions in *Homo sapiens*. The TargetScan database confirmed whether these regulations might also occur in *Oryctolagus cuniculus*. This tool enabled us to verify if the predicted binding sites were conserved in *Oryctolagus cuniculus.* Genes engaged in RHD, ALF, and MOF processes were selected. Each miR–3′-UTR interaction was checked independently. The TargetScan analysis revealed that 25 out of 40 genes for ocu-miR-21-5p, −30 out of 46 genes for ocu-miR-16b-5p, 12 out of 36 genes for ocu-miR-34-5p, 11 out of 38 genes for ocu-miR-122-5p, and 10 out of 36 genes for ocu-miR-132-5p have binding sites in 3′-UTR in *Oryctolagus cuniculus* genes. Selected miRs/target genes are presented in Table 1.

**Table 1:** Selected studied miRs/target genes involved in apoptosis and oxidative stress.

miRs	Target genes	Gene product	References used to select target genes
**Apoptosis**			
miR-21	*PTEN*	Phosphatase and tensin homolog	Buscaglia and Li (2011), Wei et al. (2013), Ghafouri-Fard et al. (2021), and He et al. (2021)
	*PDCD4*	Programmed cell death factor 4	Qiu et al. (2013) and He et al. (2021)
miR-16b	*Bcl-2*	B-cell lymphoma 2	Cimmino et al. (2005), Wu et al. (2011), Gao and Zhao (2020) and Hukowska-Szematowicz et al. (2020)
	*CXCL10*	C-X-C motif chemokine ligand 10	Gao and Zhao (2020) and Lai et al. (2021)
miR-34a	*SIRT1*	Silent information regulator 1	Yamakuchi and Lowenstein (2009), Hermeking (2010), Sharma et al. (2018), and Jiang et al. (2019)
	*p53*	Protein *p53*	
**Oxidative stress**			
miR-122	*Bach1*	BTB domain and CNC homolog 1	Shan et al. (2007), Klieser et al. (2019), and Hukowska-Szematowicz et al. (2020)
miR-132	*Nrf-2*	Nuclear factor erythroid 2-related factor 2	Wasik et al. (2017), Zhou et al. (2020), Herengt et al. (2021), Xu et al. (2021), and Mao et al. (2022)

### 2.6 miRs and mRNA isolation from tissues

Total RNA, including miRs, was extracted from 50 mg of the infected and healthy rabbits’ liver, lung, kidney, and spleen tissues using the miRNeasy Mini Kit (Qiagen, Hilden, Germany) following the manufacturer’s protocol. RNA concentration and quality were determined using a NanoDrop 2000 spectrophotometer (Thermo Fisher Scientific, Waltham, MA, United States).

### 2.7 miRs polyadenylation and reverse transcription reaction

The reverse transcription (RT) reaction was performed using a miRCURY LNA RT Kit (Qiagen, Hilden, Germany) according to the manufacturer’s instructions. In the tissue samples, 5 ng/μL of total RNA was used for cDNA synthesis. The cycling conditions for the RT reaction were as follows: incubation for 60 min at 42°C, heat inactivation of the reverse transcriptase for 5 min at 95°C, and immediate cooling to 4°C. The obtained cDNA was stored at −20°C until further experiments.

### 2.8 mRNA polyadenylation and reverse transcription reaction

cDNA synthesis was carried out using the RevertAid First Strand cDNA Synthesis Kit (Thermo Fisher Scientific, United States) according to the manufacturer’s protocol. Oligo(dT) and random hexamers were used to perform the reaction to increase its yield. The cycling conditions for the RT reaction were as follows: incubation for 5 min at 25°C followed by 60 min at 42°C, termination of the reaction by heating at 70°C for 5 min, and immediate cooling to 4°C. The obtained cDNA was stored at −20°C until further experiments.

### 2.9 Quantification of miRs in tissue samples using quantitative real-time PCR and data analysis

The expression of miRs (Table 2) was determined by the quantitative real-time PCR (qPCR) reaction in tissue samples using the miRCURY LNA miRNA PCR Assay (Qiagen, Hilden, Germany) and the miRCURY LNA SYBR Green PCR Kit, according to the manufacturer’s instructions. cDNA templates were diluted 60-fold in RNase-free water. The amplification of the selected miRs was performed using a real-time PCR system. The qPCR data were normalized using ocu-miR-103a-3p as a stable reference gene. This gene was chosen based on a previous study (Hukowska-Szematowicz et al., 2020) and evaluation under experimental conditions using the geNorm, NormFinder, and BestKeeper algorithms (Hukowska-Szematowicz et al., 2020). Fluorescence data were analyzed using a real-time PCR system, and the expression of miRs, normalized to an endogenous reference, was determined using the 2^−ΔΔCt^ formula.

**Table 2:** Sequences of the tested miRs of *Oryctolagus cuniculus* (ocu).

miRNAs	Sequence
**miRNAs tested**	
ocu-miR-21-5p	5′-UAGCUUAUCAGACUGAUGUUGACU-3′
ocu-miR-16b-5p	5′-UAGCAGCACGUAAAUAUUGGCGU-3′
ocu-miR-34a-5p	5′-UGGCAGUGUCUUAGCUGGUUGU-3′
ocu-miR-122-5p	5′-UGGAGUGUGACAAUGGUGUUUG-3′
ocu-miR-132-5p	5′-ACCAUGGCUUUCGAUUGUUACU-3′
**reference miRNAs**	
ocu-miR-103a-3p	5′-AGCAGCAUUGUACAGGGCUAUGA-3′

### 2.10 Quantification of mRNAs in tissue samples using quantitative real-time PCR and data analysis

Computational tools for online Primer-BLAST (2024) and Beacon Designer (2024) were used to design specific primers for qPCR analysis for the tested target genes (Table 3). To validate the designed primers, temperature gradient PCR was performed using the Color OptiTaq PCR Master Mix (2×) kit (Euryx, Poland), followed by agarose gel electrophoresis. It allowed us to select the optimal annealing temperature of the primers and check whether non-specific products were formed. After checking the primers, optimization of the real-time PCR reaction was performed. An optimized approach was used to sequentially optimize primer sequences, annealing temperatures, primer concentrations, and a range of cDNA concentrations for each gene tested. Using the calibration method and the standard curve allowed us to obtain a standard curve. Using the optimal annealing temperature and primer concentration for each primer pair, we used serial dilutions of the same cDNA (1:2, 1:4, 1:8, 1:16, and 1:32 dilutions). Dilutions were made from a 10 ng/μL concentration, which was recommended as the highest possible concentration by the manufacturing protocol (HOT FIREPol^®^ EvaGreen^®^ qPCR Supermix, 5×; Solis BioDyne, Estonia). We noticed that different primer pairs had different optimal cDNA concentration ranges for each gene, giving rise to the most significant coefficient of determination (R2) and optimal efficiencies (100% ± 5%). In addition, the specificity of the primers was verified experimentally by melting curve analysis. According to the manufacturer’s instructions, the qPCR reaction in tissue samples determined the mRNA expression using the HOT FIREPol^®^ EvaGreen^®^ qPCR Supermix, 5× (Solis BioDyne, Estonia). The amplification of the selected mRNAs was performed using the Quant Studio 5 Real-Time PCR system (Applied Biosystems, United States). The qPCR data were normalized using the reference gene 18S. Fluorescence data were analyzed using a real-time PCR system, and the expression of mRNA, normalized to an endogenous reference, was determined using the 2^−ΔΔCt^ formula. A melting curve analysis was performed each time.

**Table 3:** qPCR primers used in the study target gene expression.

Gene	GenBank accession no.	Primers		T_a_ (°C)	Amplicon length (bp)	T_m_ of the amplification products (°C)
		Forward	Reverse			
*PTEN*	XM_008270133.3	5′-GCGGAACTTGCAATCCTCAG-3′	5′-TCGTGTGGGTCCTGAATTGG-3′	60	77	81
*PDCD4*	XM_017348553.2	5′-GAATAACCGTGCCAACCAGTCC-3′	5′-CTTTCCCTCCTGCACCACCTTTC-3′	60	102	85
*Bcl-2*	XM_008261439.2	5′-TGTGTGTGGAGAGCGTCAAC-3′	5′-AGTTCCACGAAGGCATCCCAG-3′	62	133	87.5
*CXCL10*	XM_002717106.4	5′-AGCATTTAGCAAGGAAAGGTCCAG-3′	5′-AGAAGGGAAGTGTGGCAGAGG-3′	60	110	83.8
*p53*	XM_008270660.3	5′-TGACGGAAGTTGTCAGACGC-3′	5′-TACAGTCAGAGCCAACCTCGG-3′	60	183	89.8
*SIRT1*	XM_002718460.4	5′-AGTAAGCGGCTCGATGGTAATCAG-3′	5′-TCCAGTTCCTCCAGGTCTCTCTG-3′	63	249	85.5
*Bach1*	XM_002716782.4	5′-ACTCTACCAGAAGAGGTGACAG-3′	5′-TGAGAAACTGAAAGCAGGACTC-3′	60	160	78
*Nrf-2*	XM_051849401.1	5′-AGAAACAGAACACAAGGACATGG-3′	5′-TTGGGCTGGCTGAATTGGG-3′	60	241	85.7
*Bax*	XM_008252361.3	5′-ACATGGAGCTACAGAGGATGATCG-3′	5′-AGCGTCCAGCCCATAATAGTCC-3′	61	205	86
*Caspase-3*	NM_001082117.1	5′-AACTTTTCATTATTCAGGCTTGCCG-3′	5′-TCAACCCCACTGTCTGTCTCG-3′	58	70	84.2
*PARP*	XM_008268352.3	5′-CGGACAAGCTCTACCGAGTG-3′	5′-CATCGAACATGGGCGACTGC-3′	60	123	88
*HO-I*	XM_051846030.1	5′-ACTGCCGAGGGTTTTAAGCTGG-3′	5′-ACCGGGTTCTCCTTGTTGTGC-3′	60	92	85
*18S*	NR_033238.1	5′-ATCAGATACCGTCGTAGTTC-3′	5′-TTCCGTCAATTCCTTTAAG-3′	60	167	88

### 2.11 Statistical analysis

All results were statistically analyzed using STATISTICA PL Version 13. The Shapiro–Wilk test was performed to determine the distribution of the analyzed variables. Depending on the obtained distribution, the Student’s *t*-test for data with a normal distribution and the Mann–Whitney *U* test for non-parametric data were used. The one-way ANOVA was performed for parametric data to determine possible changes in all miRs or mRNAs; however, the Kruskal–Wallis test was used for non-parametric data. The data are presented in graphs as average values ± standard error of the mean (SEM). The values for which the *p*-value does not exceed 5% (*p* ≤ 0.05) were considered significant statistical differences. Correlation analyses were performed using the non-parametric Spearman’s rank method. Results were considered statistically significant if *p <* 0.05.

## 3 Results

### 3.1 Clinical signs of disease and post-mortem analysis

Animals infected with both *L. europaeus* genotypes—GI.1 and GI.2—showed clinical signs consistent with RHD (apathy, dyspnea, body temperature > 41°C, anorexia, and neurological symptoms). Two rabbits after the *L. europaeus* GI.2 infection died asymptomatically. Mortality after infection with *L. europaeus* in both genotypes was 90% to 60 h p.i. The *L. europaeus* GI.2 genotype was more virulent, causing 90% mortality in rabbits within 32 h p.i. and a fulminant course of the disease. The disease ranged from per-acute to acute in animals infected with this *L. europaeus* genotype. Whereas, after *L. europaeus* GI.1 infection, the mortality rate was −10% to 32 h p.i., 40% to 36 h p.i., and 40% between 56 and 60 h p.i.

### 3.2 miRs expression levels and its downstream targets involved in apoptosis during *Lagovirus europaeus* GI.1 and GI.2 genotype infection in rabbits

We analyzed the expression of miRs involved in apoptosis (miR-21, miR-34a, and miR-16b) in four tissues (liver, lung, spleen, and kidney) in rabbits infected with *L. europaeus* GI.1 and GI.2 genotypes and its downstream targets.

**Figure 1:**
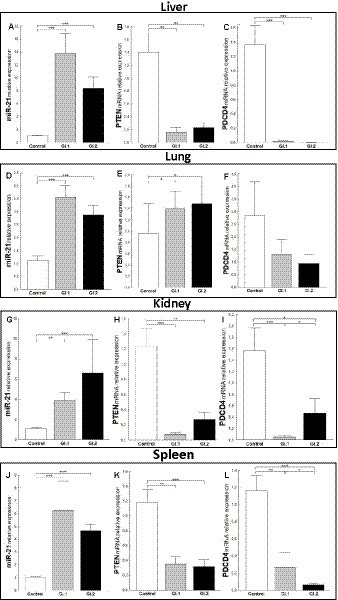
Expression of miR-21, *PTEN*, and *PDCD4* mRNA in four tested tissues during infection with *Lagovirus europaeus—*two genotypes (GI.1 and GI.2). Relative expression levels of miR-21 **(A,D,G,J)**, *PTEN* **(B,E,H,K)**, and *PDCD4* **(C,F,I,L)** in the liver **(A–C)**, lung **(D–F)**, kidney **(G–I)**, and spleen **(J–L)** of controls (*n* = 10), GI.1 (*n* = 10), and GI.2 (*n* = 10). The expression of all genes is normalized to an endogenous reference (miR-103a for miR-21 and 18S rRNA for other genes) and presented as a relative fold change to controls according to the comparative Ct method (2^−ΔΔCt^). The miR and target gene levels were evaluated using real-time PCR. Data were compared with the one-way ANOVA test or the ANOVA Kruskal–Wallis test. The *t*-test, or Mann–Whitney *U* test, was performed to assess the differences in parameter concentrations. *p*-values below 0.05 were considered statistically significant. Bars indicate the mean ± standard error of the mean (SEM), ^*^p < 0.5, ^**^p < 0.01, and ^***^p < 0.001.

Increased miR-21 expression was observed after infection with *L. europaeus*—two genotypes (GI.1 and GI.2) in all four tissues examined. In the liver, miR-21 expression was substantially increased in both *L. europaeus* GI.1 (13.2-fold changes vs. control, *p* = 0.0002) and GI.2 p.i. (8.4-fold changes vs. control, *p* = 0.0002; Figure 1A). In cases of infection with GI.1 and GI.2, upregulation of miR-21 was accompanied by decreased levels of *PTEN* (8.5-fold reduction, 88% reduction, vs. control, *p* = 0.001 and 6-fold reduction, 84% reduction, vs. control, *p* = 0.002, respectively; Figure 1B) and *PDCD4* (71.4-fold reduction, 98.6% reduction, *p* = 0.0002 and 273.4-fold reduction, 99.6% reduction, *p* = 0.0001, respectively; Figure 1C). In the lung, we also observed the upregulation of miR-21 for both genotypes compared to the control group (3.2-fold change, *p <* 0.0001 for GI.1 and 2.6-fold change, *p* = 0.0003 for GI.2; Figure 1D). In contrast to the liver, in lung tissue compared to the control, the level of *PTEN* was upregulated in GI.1 and GI.2 (1.4-fold change, *p* = 0.04 and 1.5-fold change, *p* = 0.03, respectively; Figure 1E). While no changes in *PDCD4* expression levels were noted (Figure 1F). The miR-21 level was significantly increased also in the kidneys (Figure 1G) and spleen (Figure 1J) of the rabbits infected with both *L. europaeus* genotypes, with a 3.6-fold (*p* = 0.002) and 6.1-fold (*p* = 0.0006) increase, respectively, vs. control in kidneys and a 6.1-fold change (*p* = 0.0002) and a 4.5-fold change (*p* = 0.0002), respectively, in spleen. Simultaneously, in both tissues, downregulation of *PTEN* [15.6-fold reduction, 94% reduction, *p* = 0.0002 in kidney (Figure 1H) and 3.4-fold reduction, 70.5% reduction, *p* = 0.002 in spleen (Figure 1K)] and *PDCD4* [30.4-fold reduction, 97% reduction, *p* = 0.0002 in kidney (Figure 1I) and 4.4-fold reduction, 23% reduction, *p* = 0.002 in spleen (Figure 1L)] was detected during GI.1 infection. Furthermore, during infection with the second *L. europaeus* GI.2 genotype, the expression of *PTEN* and *PDCD4* was downregulated in the kidneys and spleen compared to healthy rabbits. *PTEN* [4.6-fold reduction, 78% reduction, *p* = 0.002 in kidney (Figure 1H)] and 3.7-fold reduction, 73% reduction, *p* = 0.0003 in spleen (Figure 1K). *PDCD4* [3.3-fold reduction, 70% reduction, *p* = 0.01 in kidney (Figure 1I) and 18.7-fold reduction, 95% reduction, *p* = 0.0002 in spleen (Figure 1L)].

**Figure 2:**
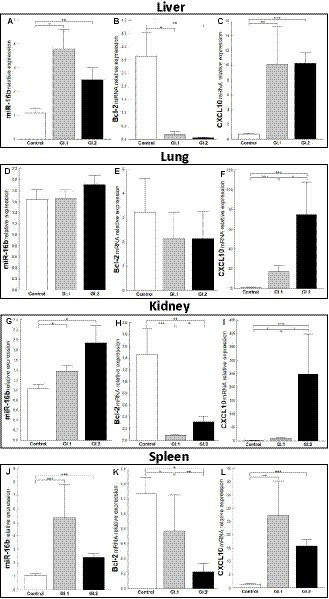
Expression of miR-16b, *Bcl-2*, and *CXCL10* mRNA in four tissues during infection with *Lagovirus europaeus*—two genotypes (GI.1 and GI.2). Relative expression levels of miR-16b **(A,D,G,J)**, *Bcl-2* **(B,E,H,K)**, and *CXCL10* **(C,F,I,L)** in the liver **(A–C)**, lung **(D–F)**, kidney **(G–I)**, and spleen **(J–L)** of controls (*n* = 10), GI.1 (*n* = 10), and GI.2 (*n* = 10). The expression of all genes is normalized to an endogenous reference (miR-103a for miR-16b and 18S rRNA for other genes) and presented as a relative fold change to controls according to the comparative Ct method (2^−ΔΔCt^). The miR and target gene levels were evaluated using real-time PCR. Data were compared with the one-way ANOVA test or the ANOVA Kruskal–Wallis test. The *t*-test, or Mann–Whitney *U* test, was performed to assess the differences in parameter concentrations. *p*-values below 0.05 were considered statistically significant. Bars indicate the mean ± standard error of the mean (SEM), ^*^p < 0.5, ^**^p < 0.01, and ^***^p < 0.001.

The expression level of miR-16b was significantly higher in tissues (liver, kidney, and spleen) in both infected groups of rabbits, excluding the lungs. In the liver, we observed a 3.5-fold change, *p* = 0.002 for GI.1, and a 2.3-fold change, *p* = 0.01 for GI.2 (Figure 2A). The expression of miR-16b in the lung was unchanged (Figure 2D). In the kidneys, the expression of miR-16b for GI.1 and GI.2 increased 1.3-fold, *p* = 0.023, and 1.9-fold, *p* = 0.19, respectively (Figure 2G). Whereas in the spleen during *L. europaeus* GI.1 infection, we observed the highest increase in miR-16b levels, which was a 5-fold change (*p* = 0.0002), while during GI.2, expression was 2.2-fold higher (*p* = 0.0003) compared to the control (Figure 2J).

Upregulation of miR-16b was associated with downregulation of Bcl-2 in both infected groups compared to controls in the liver (Figure 2B), kidneys (Figure 2H), and spleen (Figure 2K). However, the expression of *Bcl-2* mRNA in the lung was unchanged (Figure 2E). In liver tissue, the reduction of *Bcl-2* was 16.2-fold, 94% reduction, *p* = 0.02 for GI.1 and 49.4-fold, 98% reduction, *p* = 0.004 for GI.2 (Figure 2B). For kidney tissue, the reduction was accordingly 16.7-fold reduction, 94% reduction, *p* = 0.0004 and 4.6-fold reduction, 78% reduction, *p* = 0.005 for GI.1 and GI.2 (Figure 2H). The minor decrease in *Bcl-2* expression was observed in the spleen and amounted to a 1.6-fold reduction (39% reduction, *p* = 0.03) for GI.1 and a 5.7-fold reduction (82% reduction, *p* = 0.001) for GI.2 compared to the control (Figure 2K). In the case of *CXCL10*, another target gene for miR-16b, significantly enhanced expression was noted in all tested tissues (Figures 2C,F,I,L). The highest expression was observed in the kidney during the GI.2 infection, and it amounted to a 162.6-fold change, *p* = 0.0002 (Figure 2I). On the other hand, the lowest increase in expression was also in the kidney, but in the *L. europaeus* GI.1-infected group, it amounted to a 6.2-fold change, *p* = 0.03 compared to the control (Figure 2I).

**Figure 3:**
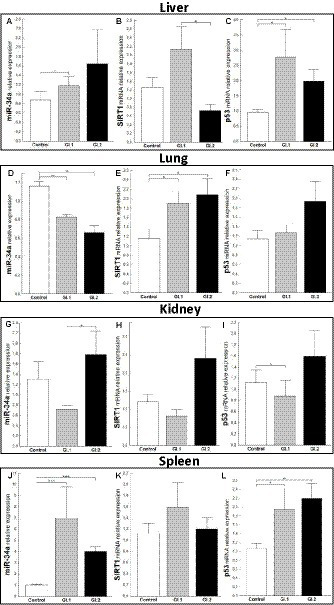
Expression of miR-34a, *SIRT1*, and *p53* mRNA in four tissues during infection with *Lagovirus europaeus*—two genotypes (GI.1 and GI.2). Relative expression levels of miR-34a **(A,D,G,J)**, *SIRT1* **(B,E,H,K)**, and *p53* **(C,F,I,L)** in the liver **(A–C)**, lung **(D–F)**, kidney **(G–I)**, and spleen **(J–L)** of controls (*n* = 10), GI.1 (*n* = 10), and GI.2 (*n* = 10). The expression of all genes is normalized to an endogenous reference (miR-103a for miR-34a and 18S rRNA for other genes) and presented as a relative fold change to controls according to the comparative Ct method (2^−ΔΔCt^). The miR and target gene levels were evaluated using real-time PCR. Data were compared with the one-way ANOVA test or the ANOVA Kruskal–Wallis test. The *t*-test, or Mann–Whitney *U* test, was performed to assess the differences in parameter concentrations. *p*-values below 0.05 were considered statistically significant. Bars indicate the mean ± standard error of the mean (SEM), ^*^p < 0.5, ^**^p < 0.01, and ^***^p < 0.001.

In the case of miR-34a in the liver, only a change in expression was demonstrated during *L. europaeus* GI.1 genotype infection and was upregulated by a 1.6-fold change, *p* = 0.04 (Figure 3A). Whereas, no statistically significant change was observed in the expression level of *SIRT1*, a downstream target of miR-34a, in both of the infected groups of rabbits (Figure 3B). However, a significant increase in *p53*, a transcriptional activator of miR-34a, was observed in infected rabbits compared to controls [2.9-fold change, *p* = 0.02 for GI.1 vs. control and 2.1-fold change, *p* = 0.01 for GI.2 vs. control (Figure 3C)]. Our research demonstrated that in the lung, miR-34a was significantly suppressed in the GI.1 (1.4-fold reduction, 29% reduction, *p* = 0.003) and GI.2 (1.8-fold reduction, 43% reduction, *p* = 0.003) groups (Figure 3D). In both of the infected groups of rabbits (GI.1 and GI.2), downregulation of miR-34a was accompanied by an increase in *SIRT1* (1.6-fold change, *p* = 0.03 and 1.8-fold change, *p* = 0.029, respectively; Figure 3E). However, no change in *p53* expression was observed in either group (Figure 3F). Compared to the control, the level of miR-34a and *SIRT1* was unchanged in the kidney tissue (Figures 3G,H) from both of the infected groups of rabbits. The only change in the kidneys was observed in the level of *p53* during infection with *L. europaeus* GI.1 (Figure 3I). The level of *p53* decreased 1.3-fold (22% reduction, *p* = 0.04 vs. control). In the spleen, it was noted that there was an increase in miR-34a levels in both *L. europaeus* genotypes (6.8-fold change, *p* = 0.0002 in the GI.1 group and 3.9-fold change, *p* < 0.0001 in the GI.2 group; Figure 3J) and increased expression of *p53* (1.9-fold change, *p* = 0.04 for the GI.1 vs. control and 2.2-fold change, *p* = 0.004 for the GI.2 vs. control; Figure 3L). However, no change in expression was detected for the miR-34a target gene—*SIRT1* (Figure 3K).

### 3.3 Biomarkers of apoptosis *Bax*, *Bax*/*Bcl-2* ratio, *Caspase-3*, and *PARP* during *Lagovirus europaeus* GI.1 and GI.2 genotype infection in rabbits

The study aimed to determine the relative level of mRNA expression of proapoptotic biomarkers of apoptosis—*Bax*, *Bax*/*Bcl-2* ratio, *Caspase-3*, and *PARP*—to assess the degree of apoptosis in the liver, lung, kidney, and spleen.

**Figure 4:**
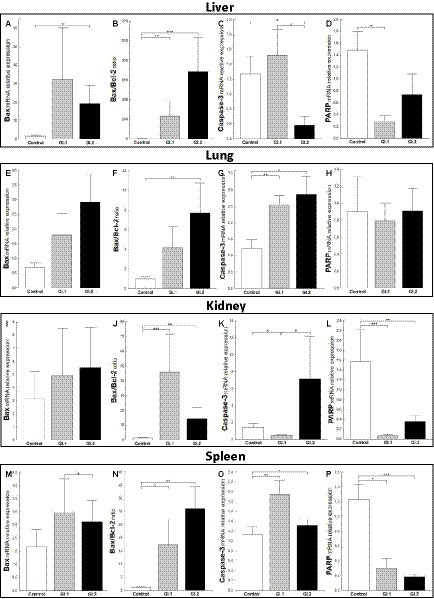
Expression levels of biomarkers *Bax* mRNA, *Bax/Bcl-2* ratio, Caspase-3, and *PARP* mRNA in four tissues during infection with *L. europaeus*—two genotypes (GI.1 and GI.2). Relative expression levels of *Bax* **(A,E,I,M)**, *Bax/Bcl-2* ratio **(B,F,J,N)**, Caspase-3 **(C,G,K,O)**, and PARP **(D,H,L,P)** in the liver **(A–D)**, lung **(E–H)**, kidney **(I–L)**, and spleen **(M–P)** of controls (*n* = 10), GI.1 (*n* = 10), and GI.2 (*n* = 10). The expression of the gene is normalized to an endogenous reference 18S rRNA and presented as a relative fold change to controls according to the comparative Ct method (2^−ΔΔCt^). The mRNA levels were evaluated using real-time PCR. Data were compared with the one-way ANOVA test or the ANOVA Kruskal–Wallis test. The *t*-test, or Mann–Whitney *U* test, was performed to assess the differences in parameter concentrations. *p*-values below 0.05 were considered statistically significant. Bars indicate the mean ± standard error of the mean (SEM), ^*^p < 0.5, ^**^p < 0.01, and ^***^p < 0.001.

The relative level of mRNA expression of *Bax* was unchanged in most examined tissues except liver tissue during *L. europaeus* GI.2 infection and was 12.3-fold higher compared to control (*p* = 0.03; Figures 4A,E,I,M). However, a *Bax*/*Bcl-2* ratio mRNA was largely changed and was the highest in the liver, where upregulation was 181-fold (*p* = 0.006 vs. control) and 535-fold (*p* = 0.0003 vs. control) for GI.1 and GI.2, respectively (Figure 4B). In the lungs, an increase in the *Bax*/*Bcl-2* ratio was found only in the GI.2 group (7.9-fold, *p* = 0.01 vs. control; Figure 4F). Compared to the control, the *Bax*/*Bcl-2* ratio was 35.6-fold enhanced for GI.1 (*p* = 0.0007) and 11.2-fold enhanced for GI.2 (*p* = 0.005) in the kidneys (Figure 4J). In turn, in the spleen, we observed an upregulation of *Bax*/*Bcl-2* ratio of 15.8-fold level growth (*p* = 0.02) in the GI.1 group and 28.3-fold level growth (*p* = 0.003) in the GI.2 group compared to tissues from healthy rabbits (Figure 4N).

Caspases are cysteine proteases that play fundamental roles in the apoptotic responses of cells to different stimuli. Our research indicated downregulation of relative levels of *Caspase-3* mRNA in the liver in the GI.2-infected group (2.2-fold reduction, 54% reduction, *p* = 0.02; Figure 4C) and the kidney in the GI.2-infected group (2.9-fold reduction, 66% reduction, *p* = 0.02; Figure 4K). Whereas, in the lung and spleen, we found an upregulation of the expression of Caspase-3 for both viruses. During infection with the *L. europaeus* GI.1 genotype, we noted a similar increase in the expression level of *Caspase-3* in the lung and in the spleen, which was a 2.1-fold change (*p* = 0.003 for the lung; Figure 4G *p* = 0.007 for spleen; Figure 4O) compared to control. Whereas during *L. europaeus* GI.2 infection, in the lung level of *Caspase-3* enhanced 2.3-fold change (*p* = 0.016 vs. control; Figure 4G) and in the spleen 1.4-fold change (*p* = 0.04 vs. control; Figure 4O). In other cases (liver in the GI.1 group (Figure 4C) and kidney in the GI.2 group (Figure 4K), the level of *Caspase-3* was unchanged).

Furthermore, we examined the level of *PARP* mRNA expression. We observed downregulation of *PARP* in the liver of rabbits infected with *L. europaeus* GI.1 (5.4-fold reduction, 81% reduction, *p* = 0.004 vs. control; Figure 4D). While in the liver in the GI.2 group, expression of *PARP* was unchanged (Figure 4D). We also noted unchanged *PARP* levels in the lungs during infection with both *L. europaeus* genotypes (Figure 4H). However, the expression of *PARP* was significantly decreased in the kidneys and spleen of the rabbits infected with the *L. europaeus* GI.1 and GI.2 genotypes, with a 22.6-fold reduction (95% reduction, *p* = 0.0004 vs. control) and a 4.4-fold reduction (77% reduction, *p* = 0.006 vs. control), respectively, in the kidney (Figure 4L), and a 4.1-fold reduction (76% reduction, *p* = 0.002 vs. control) and a 6.7-fold reduction (85% reduction, *p* = 0.0006 vs. control), respectively, in the spleen (Figure 4P).

### 3.4 miR expression levels and its downstream targets involved in oxidative stress during *Lagovirus europaeus* GI.1 and GI.2 infection in rabbits

We further investigated the expression of miRs and target genes involved in oxidative stress—miR-122 (*Bach1*) and miR-132 (*Nrf-2*).

**Figure 5:**
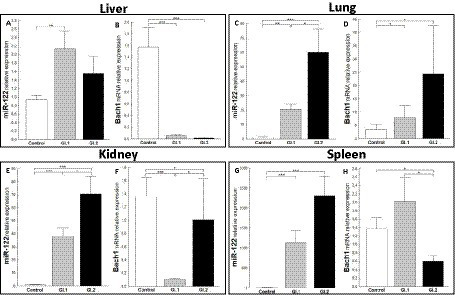
Expression of miR-122 and *Bach1* mRNA in four tissues during infection with *L. europaeus*—two genotypes (GI.1 and GI.2). Relative expression levels of miR-122 **(A,C,E,G)** and *Bach1* **(B,D,F,H)** in the liver **(A,B)**, lung **(C,D)**, kidney **(E,F)**, and spleen **(G,H)** of controls (*n* = 10), GI.1 (*n* = 10), and GI.2 (*n* = 10). The expression of all genes is normalized to an endogenous reference (miR-103a for miR-122 and 18S rRNA for other genes) and presented as a relative fold change to controls according to the comparative Ct method (2^−ΔΔCt^). The miR and target gene levels were evaluated using real-time PCR. Data were compared with the one-way ANOVA test or the ANOVA Kruskal–Wallis test. The *t*-test, or Mann–Whitney *U* test, was performed to assess the differences in parameter concentrations. *p*-values below 0.05 were considered statistically significant. Bars indicate the mean ± standard error of the mean (SEM), ^*^p < 0.5, ^**^p < 0.01, and ^***^p < 0.001.

The relative expression of miR-122 was significantly different in the liver tissue of the infected rabbits compared to the healthy rabbits only during infection with *L. europaeus* GI.1 (2.3-fold change, *p* = 0.002; Figure 5A). While in liver tissue, a downregulation of *Bach1* was noted for both infected groups (27.6-fold reduction, 96% reduction, *p* = 0.0002 for GI.1 and 98-fold reduction, 99% reduction, *p* = 0.00018 for GI.2; Figure 5B). In the lung, we observed the upregulation of miR-122 for both *L. europaeus* genotypes compared to the control group (17.1-fold change, *p* = 0.001 for GI.1 and 49.4-fold change, *p* = 0.0007 for GI.2; Figure 5C) with a simultaneous increase in expression of *Bach1* (2.3-fold change; *p* = 0.04 and 7.1-fold change, *p* = 0.02 for GI.1 and GI.2, respectively; Figure 5D). The miR-122 level was significantly increased in the kidneys of the rabbits infected with GI.1 and GI.2, with a 34.8-fold (*p <* 0.0001) and 64.3-fold (*p* = 0.0001) increase, respectively (Figure 5E). The upregulation of miR-122 was accompanied by significantly decreased *Bach1* in both infected groups (13.6-fold reduction, 93% reduction, *p* = 0.0003 in the GI.1 group and 1.4-fold reduction, 26% reduction, *p* = 0.04 in the GI.2 group; Figure 5F). While the highest increase in miR-122 expression occurred in the spleen and was, respectively, 554.3-fold change (*p* = 0.0002) for GI.1 and 1,135-fold change (*p* = 0.0002) for GI.2 (Figure 5G). However, a change in *Bach1* expression in the spleen was noted only in infection with GI.2 and was reduced by 2.3-fold (56% reduction, *p* = 0.02 vs. control; Figure 5H).

**Figure 6:**
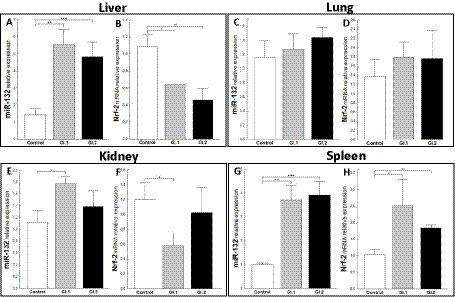
Expression of miR-132 and *Nrf-2* mRNA in four tissues during infection with *L. europaeus*—two genotypes (GI.1 and GI.2). Relative expression levels of miR-132 **(A,C,E,G)** and *Nrf-2* **(B,D,F,H)** in the liver **(A,B)**, lung **(C,D)**, kidney **(E,F)**, and spleen **(G,H)** of controls (*n* = 10), GI.1 (*n* = 10), and GI.2 (*n* = 10). The expression of all genes is normalized to an endogenous reference (miR-103a for miR-132 and 18S rRNA for other genes) and presented as a relative fold change to controls according to the comparative Ct method (2^−ΔΔCt^). The miR and target gene levels were evaluated using real-time PCR. Data were compared with the one-way ANOVA test or the ANOVA Kruskal–Wallis test. The *t*-test, or Mann–Whitney *U* test, was performed to assess the differences in parameter concentrations. *p*-values below 0.05 were considered statistically significant. Bars indicate the mean ± standard error of the mean (SEM), ^*^p < 0.5, ^**^p < 0.01, and ^***^p < 0.001.

An extensively upregulated miR-132 expression was noted in the liver (3.9-fold, *p* = 0.0003 for GI.1 and 3.4-fold, *p* = 0.002 for GI.2; Figure 6A) and spleen (3.6-fold, *p* = 0.002 and 3.9-fold, *p <* 0.0001; Figure 6G) during viral infection compared to the control group. In the liver, where expression of miR-132 was increased, the expression of *Nrf-2*, a critical factor in antioxidant defense, was inhibited in comparison to controls in both infected groups, with a 1.7-fold reduction (41% reduction, *p* = 0.02) for GI.1 and a 2.4-fold reduction (58% reduction, *p* = 0.007) for GI.2 (Figure 6B). In the lung in both infected groups, no change was demonstrated in levels of miR-132 and *Nrf-2* (Figures 6C,D). The same observations as for the lungs were made for kidney tissue in the GI.2 group (Figures 6E,F). In the kidneys, an increase in miR-132 expression only occurred during GI.1 infection (1.6-fold change, *p* = 0.02; Figure 6E). Whereas in the kidney in the GI.1 group, the expression of *Nrf-2* was inhibited 2-fold (50% reduction, *p* = 0.025 vs. control; Figure 6F). In the spleen, an increase in miR-132 expression was accompanied by an increase in *Nrf-2* expression for both *L. europaeus* genotypes (2.4-fold change, *p* = 0.02 for GI.1 and 1.8-fold change, *p* = 0.008 for GI.2) compared to healthy rabbits (Figure 6H).

### 3.5 *HO-I* as a biomarker of the response to oxidative stress during *Lagovirus europaeus* GI.1 and GI.2 genotype infection in rabbits

**Figure 7:**
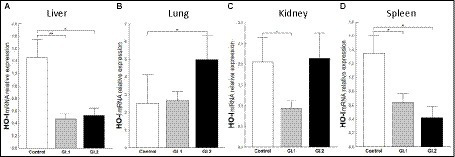
Expression of biomarker *HO-I* mRNA in four tissues during infection with *Lagovirus europaeus*—two genotypes (GI.1 and GI.2). Relative expression levels of *HO-I* **(A–D)** in the liver **(A)**, lung **(B)**, kidney **(C)**, and spleen **(D)** of controls (*n* = 10), GI.1 (*n* = 10), and GI.2 (*n* = 10). The expression of the gene is normalized to an endogenous reference 18S rRNA and presented as a relative fold change to controls according to the comparative Ct method (2^−ΔΔCt^). The mRNA levels were evaluated using real-time PCR. Data were compared with the one-way ANOVA test or the ANOVA Kruskal–Wallis test. The *t*-test, or Mann–Whitney *U* test, was performed to assess the differences in parameter concentrations. *p*-values below 0.05 were considered statistically significant. Bars indicate the mean ± standard error of the mean (SEM), ^*^p < 0.5, ^**^p < 0.01, and ^***^p < 0.001.

To assess the antioxidative response of *Bach1* and *Nrf-2* during *L. europaeus* GI.1 and GI.2 genotype infection in rabbits, we estimated their effect on the downstream target gene *HO-I*. We found that *HO-I* levels were 3.1-fold (67% reduction, *p* = 0.005 vs. control) lower in livers from rabbits infected with *L. europaeus* GI.1 and 2.8-fold (64% reduction, *p* = 0.026 vs. control) lower in livers from rabbits infected with GI.2 (Figure 7A). In the kidney, inhibition of *HO-I* was observed only during *L. europaeus* GI.1 infection (2.4-fold reduction, 58% reduction, *p* = 0.02; Figure 7C). Moreover, *HO-I* mRNA expression levels were 2.1-fold lower (52% reduction, *p* = 0.02) for the GI.1 group and 3.2-fold lower (69% reduction, *p* = 0.045) for the GI.2 group than in controls in the spleen (Figure 7D). However, in the lung in group GI.2, we observed an upregulation as a 2-fold change (*p* = 0.02 vs. control; Figure 7B) of *HO-I*.

## 4 Discussion

Little is known about the molecular signatures of regulatory interactions between specific miRs, apoptosis, and oxidative stress in viral diseases. We were the first to examine the expression of three miRs with proapoptotic (miR-16b, miR-34a) and antiapoptotic (miR-21) effects, which regulate both the intrinsic and extrinsic apoptosis pathways, to determine their regulatory effects on target genes and their potential impact on the pathways in infection with *L. europaeus*. In the case of oxidative stress, we studied miR-122 and miR-132 and their target genes. To investigate apoptosis and oxidative stress as a response to the viral stimulus, we examined apoptosis biomarkers (*Bax, Bax/Bcl-2 ratio, Caspase-3, PARP*) and oxidative stress (*HO-I*).

Bcl-2 family proteins are important regulatory molecules of apoptosis. Members of this family can be divided into the antiapoptotic family, which includes Bcl-2, and the apoptotic family, which includes Bax. The ratio of antiapoptotic Bcl-2 to proapoptotic Bax contributes to the susceptibility of a given cell to apoptosis (San-Miguel et al., 2006). After infection of rabbits with *L. europaeus* GI.1 and GI.2 genotypes, there is an increase in the expression of the *Bax* gene, which promotes programmed cell death (PCD). The *Bax* biomarker increases as much as 12.3-fold in the liver of rabbits during *L. europaeus* GI.2 infection, which indicates rapid (32 h p.i.) and extensive apoptosis of hepatocytes in this organ in response to the viral stimulus. Moreover, Chen et al. (2018) reported increased mRNA *Bax* expression during RHDV infection in cells transfected with NSP6 at 24 and 36 h p.i. compared to the control. The same result regarding the upregulation of Bcl-2 family proteins (determined at the level of *Bax* and *Bcl-2* mRNA) in the liver tissue and in the spleen and kidneys after infection of rabbits with the *L. europaeus* GI.2 genotype was obtained by Bebnowska et al. (2023, 2024). In our lung and spleen results, the *Bax* mRNA gene expression level indicates a lower degree of apoptosis (statistically insignificant). It is difficult to interpret the lack of changes in *Bax* mRNA expression in the kidneys, and it may result from negative regulation by *p53*. Because *p53* promotes the apoptotic capacity of *Bax* as the main transcription factor, and since we have a reduction (22% reduction) of *p53* in the kidneys during *L. europaeus* GI.1 infection and no changes during GI.2 infection, such a scenario is possible. Vallejo et al. (2014) stated that after infection with *L. europaeus* GI.1 at 30 and 36 h p.i., there is a significant inhibition of the expression of Bcl-2 and Bcl-xL, two antiapoptotic proteins involved in the intrinsic apoptosis pathway.

During apoptosis, Bax protein bound to Bcl-2 protein activates a cascade of reactions by releasing cytochrome c from mitochondria, which helps in the successive activation of Caspases and ultimately leads to cell death (Pepper et al., 1997). It has been proposed (Pepper et al., 1997) that the association and ratio of Bax to Bcl-2 determine cell survival or death after an apoptotic stimulus. High Bax levels and/or a decrease in Bcl-2 and a high Bax/Bcl-2 ratio promote apoptosis (Pepper et al., 1997). We demonstrated a decrease in antiapoptotic *Bcl-2* and an increase in proapoptotic *Bax* during *L. europaeus* GI.1 and GI.2 genotype infection in four tissues of rabbits. We showed that the *Bax/Bcl-2* ratio promotes apoptosis during infection with *L. europaeus—*two genotypes—and was more strongly expressed (2–3 times) during GI.2 infection. This process included, in order, the liver (535-fold and 181-fold, respectively, GI.2 and GI.1), spleen (28.3-fold and 15.8-fold, GI.2 and GI.1), and lungs (7.9-fold GI.2). In the kidney, a different effect was observed (11.2-fold and 35.6-fold, GI.2 and GI.1). Our studies are consistent with previous observations by other authors, which were carried out after infection with *L. europaeus* GI.1 genotype and GI.2 genotype and concerned mainly the liver (San-Miguel et al., 2006; Chen et al., 2018), as well as the kidney and spleen; however, no changes were noted in the lungs and spleen (Bebnowska et al., 2023).

Caspase-3 is the main effector of Caspase in the external and internal apoptosis pathways, acting on enzymes involved in DNA fragmentation and chromatin condensation (Duprez et al., 2009). In our studies, the recorded increase in the *Caspase-3* gene at a similar level in the lungs (2.1-fold change and 2.3-fold change, respectively, GI.1 and GI.2) and in the spleen (2.1-fold change and 1.4-fold change, GI.1 and GI.2) indicates the activation of apoptosis in these organs. Garcia-Lastra et al. (2010) have shown a marked increase in Caspase-3 activity in the liver at 36 h (12.5-fold) and 48 h (12.6-fold) with RHDV-infected animals. Chen et al. (2018) found that non-structural protein 6 (NSP6)-induced Caspase-3 activity in *Oryctolagus cuniculus* kidney cells (RK13). However, other studies showed a significant increase in *Caspase-3* mRNA in rabbit liver (Bebnowska et al., 2024) and cleaved protein *Caspase-3* in lung, heart, kidney, and spleen during infection with the *L. europaeus* GI.2 genotype (Bebnowska et al., 2023). This methodological approach is better for measuring apoptosis levels/activity biomarkers (Caspase-3, PARP, and Bax/Bcl-2 proteins). On the other hand, in our own studies after infection of rabbits with *L. europaeus* GI.2, a reduction in the relative mRNA level of *Caspase-3* was observed in the liver (2.2-fold reduction, 54% reduction) and in the kidneys during *L. europaeus* GI.1 infection (2.9-fold reduction, 66% reduction). These results are consistent with the observations of Vallejo et al. (2014), who showed that apoptosis in RHD is induced by *L. europaeus* GI.1 genotype in the liver occurred without significantly decreasing Caspase-3 activity at 12 h, 18 h, and 24 h p.i. In the same research (Vallejo et al., 2014), infection of rabbits with RHDV resulted in a marked increase of Caspase-3 activity only at 30 and 36 h p.i. in the liver.

PARP is widely known as an enzyme that plays a role in DNA damage detection and repair. Activation of PARP in the event of DNA damage enables poly(ADP-ribosylation) of appropriate proteins, influencing repair systems, which helps maintain genome stability (Chaitanya et al., 2010). Inhibition of the PARP protein leads to the accumulation of DNA damage, which contributes to cell death (Chaitanya et al., 2010). Caspase-3 is primarily responsible for cleaving the 116 kDa PARP-1 protein into an 85 kDa fragment, whose presence indicates that cells are undergoing apoptosis (San-Miguel et al., 2006). Our results indicate that during *L. europaeus* infection, there is a reduction of mRNA *PARP* of 76%–95% in the liver, kidneys, and spleen (no significance was found in the lungs), which may indicate the accumulation of DNA damage and the process of apoptosis in cells. In Western blot analysis, San-Miguel et al. (2006) and Vallejo et al. (2014) demonstrated that in later periods of RHDV infection (36 h and 38 h p.i.), there was marked proteolysis of PARP-1, a nuclear enzyme whose cleavage into an 85 kDA fragment by Caspase-3 confirms that cells are undergoing apoptosis. Using the same technique, other authors (Bebnowska et al., 2023) showed elevated levels of the cleaved form of PARP in the heart, kidney, and spleen during *L. europaeus* GI.2 genotype infection in rabbits. No significant changes were observed in the lungs. The above result indicates that in the infection of rabbits with *L. europaeus*—two genotypes (GI.1 and GI.2)—cell death is activated by apoptosis in four tissues—liver, lungs, kidneys, and spleen—and it was more strongly expressed after *L. europaeus* GI.2 infection. Apoptosis was accompanied by increased expression or reduction of apoptosis biomarkers *Bax, Bax/Bcl-2 ratio, Caspase-3, and PARP*.

Our study demonstrated that miR-21, involved in the regulation of the external apoptosis pathway (Su et al., 2015), has antiapoptotic effects in all tissues tested after *L. europaeus* infection and has an inhibitory effect on the target genes *PTEN* and *PDCD4* and an effect on *Caspase-3* which is equivalent to reduced apoptosis. The exception is the lungs, where no inhibitory mechanism is observed during infection with *L. europaeus*. For this event, it can be suggested that there are other mechanisms of *PTEN* induction (one of the most important genes of the apoptosis pathway) and *PDCD4* induction that require further investigation. A similar regulation of miR-21 was observed in studies on the HBV, Chandipura virus, and hepatic stellate cells (Qiu et al., 2013; Wei et al., 2013; Fu et al., 2017; Pandey et al., 2021).

Our studies show that miR-16b, involved in regulating the intrinsic apoptosis pathway (Su et al., 2015), has a proapoptotic effect in most of the examined tissues, inhibiting *Bcl-2*, which increases apoptosis and increases *Bax* expression. This result is confirmed by previous research by Hukowska-Szematowicz et al. (2020) during *L. europaeus* GI.1 (GI.1a variant, strain Erfurt) infection in rabbits (2.5-fold change) and other studies (Su et al., 2015). Our current research excludes the lungs, where we do not observe changes in miR-16b and *Bcl-2* expression during *L. europaeus* GI.1 and GI.2 genotype infection. Moreover, we have shown that miR-16b affects the increase in the chemoattractant *CXCL10* in the liver, spleen, and kidneys, which may result in increased apoptosis and tissue damage. The exception is the lungs, where no change in miR-16b expression was observed, but an increase in *CXCL10* expression was observed in both *L. europaeus* genotypes. The increase in *CXCL10* expression differs from the literature data because no inhibitory mechanism via miR-16b is observed (Gao and Zhao, 2020). Perhaps in the case of infection with *L. europaeus*, there is an activating mechanism through miR-16b. According to one theory, miR-16b binds to the gene promoter and influences its increased transcription, which may have been reflected in our research on *CXCL10*. Our results differ from those recorded after HBV infection, where a downregulation of miR-16 in HepG2 cells was noted during transfection of HBV X protein. However, the results may differ due to the research model (*in vitro* vs. *in vivo* study) and the occurrence of acute and chronic forms of RHD during *L. europaeus* infection. A rapid course of infection may lead to faster damage to the organ and an increase in the level of miR-16 associated with apoptosis (Wu et al., 2011).

MiR-34a regulates the extrinsic apoptosis pathway, and its targets are *SIRT1* and *p53* (Yamakuchi et al., 2008; Hermeking, 2010; Su et al., 2015; Sharma et al., 2018; Jiang et al., 2019; Hao et al., 2021; Zhu et al., 2021). The literature describes a feedback mechanism for miR-34a in which miR-34a inhibits *SIRT1*, thereby increasing the expression of *p53*, which is known as a transcriptional activator of miR-34a (Yamakuchi et al., 2008; Jiang et al., 2019; Hao et al., 2021; Zhu et al., 2021). Our study demonstrated that miR-34a has a proapoptotic effect after infection of rabbits with *L. europaeus* GI.1 in the liver and after infection with *L. europaeus* GI.1 and GI.2 (in the spleen) and does not affect *SIRT1* expression but induces *p53* in these organs (GI.1 and GI.2). This induction of *p53* leads to an increase in apoptosis. This increase in apoptosis may occur because miR-34 is a transcriptional target of *p53*, suggesting a positive feedback loop between *p53* and miR-34a. A noticeable regulatory effect of miR-34a was observed in the lungs, the decrease of which induced an increase in *SIRT1* and did not alter the regulation of *p53*. Our observations are partly consistent with those of other researchers. Jiang et al. (2019) report that the coxsackie B3 virus increases the expression of miR-34a during infection, which induces cardiomyocyte apoptosis by activating the *SIRT1*-*p53* pathway. Studies (Sharma et al., 2018) also show that during T-lymphotropic virus-1 infection, there is an increase in miR-34a, which increases the level of *p53*. Additionally, researchers (Hao et al., 2021) have shown that treating infected cell lines with the *p53* activator nutlin-3a leads to a further increase in miR-34a, creating a feedback loop similar to that observed during *L. europaeus* infection in the liver and spleen with *L. europaeus* GI.1 or GI.2 genotypes, respectively. Furthermore, other factors that influence organ damage increase the expression of miR-34a and *p53*, contributing to apoptosis and increasing the *Bax/Bcl-2* ratio, similar to our studies (Sharma et al., 2018; Jiang et al., 2019; Hao et al., 2021).

Based on our previous literature review (Ostrycharz and Hukowska-Szematowicz, 2022), we indicated that miR-122 is involved in oxidative stress and regulates *Bach1*, thereby influencing the level of *HO-I* (the regulatory effect of miR-122 on the *Bach1*/*HO-I* axis;Shan et al., 2007; Qiu et al., 2010; Ostrycharz and Hukowska-Szematowicz, 2022; Wang et al., 2022). Many studies have shown that miR-122 is essential for HCV replication (Jopling et al., 2005; Jopling, 2008; Jopling et al., 2008; Amador-Canizares et al., 2018). On the other hand, literature data indicate that in the course of HCV and HBV infection in people (Ostrycharz and Hukowska-Szematowicz, 2022), there is a decrease in the expression of miR-122. It, in turn, inhibits the increase in the expression of *Bach1*, which in turn affects the increase in the expression of *HO-I*, and exerts a protective effect in this tissue microenvironment (Shan et al., 2007; Qiu et al., 2010; Ostrycharz and Hukowska-Szematowicz, 2022; Wang et al., 2022). No such effect was observed in our studies. Therefore, we propose an alternative mechanism for the action of miR-122 in the examined tissues during *L. europaeus* infection. Our studies indicate that miR-122 may participate in cell damage, and its increase in expression in almost all examined tissues (except for the lungs) decreases *Bach1*. The upregulation of miR-122, in turn, impacts the inhibition of *HO-I* activity, which can lead to increased tissue damage. Our results indicate that *HO-I* in *L. europaeus* infection has no protective effect. The association of the proposed pathway with clinical documentation indicates that the protective role of *HO-I* can only be considered in the lungs during *L. europaeus* GI.2 infection. In the case of miR-122, the highest expression of these molecules was recorded in the spleen, kidney, lung, and liver during *L. europaeus* GI.1 infection and the same tissues except the liver with GI.2 infection. The latter result is consistent with previous observations by Hukowska-Szematowicz et al. (2020) during *L. europaeus* GI.1 (GI.1a variant, strain Erfurt) infection in rabbits, which did not show miR-122 expression in the liver but in the serum.

The regulatory effect of miR-132 in the oxidative stress pathway in viral infection proposed in our study has yet to be studied. The involvement of miR-132 in oxidative stress has been confirmed in other diseases (Wasik et al., 2017; Zhou et al., 2020; Xu et al., 2021). Our studies suggested that miR-132 can be involved in tissue damage during *L. europaeus* infection. Overexpression of miR-132 during *L. europaeus* GI.1 and GI.2 infection may be an element of the pathogenesis of RHD. The conducted research showed increased miR-132 in the liver and spleen during *L. europaeus* GI.1 and GI.2 infection. Whereas in the kidney, it was noted that miR-132 expression increased only during infection with *L. europaeus* GI.1. The mechanism of *Nrf-2* inhibition via miR-132 is observed only in the liver and kidneys. This mechanism correlates with a simultaneous decrease in *HO-I* expression, which may increase liver and kidney damage. Interestingly, in the spleen, despite the increase in miR-132, an increased level of *Nrf-2* is observed with a simultaneous decrease in the level of *HO-I*. Further research is required to investigate this mechanism. Data on Nrf-2 signaling (a critical factor in oxidative defense) in viral infections are limited (Herengt et al., 2021). Evidence suggests that activation of Nfr-2 in host cells is protective during viral infections. Protection may be through either antiviral activity, inhibition of cell death to protect against excessive tissue damage, or both (Herengt et al., 2021). There was no change in miR-132 expression in the lungs after infection with *L. europaeus* GI.1 and GI.2. In the lungs, after the *L. europaeus* GI.1 infection, no *HO-I* changes were noted. It can be assumed that the result of these reactions is less oxidative damage in the lungs and more significant damage in the liver, spleen, and kidneys. Our results regarding the expression of *Nrf-2* in response to *L. europaeus* infection confirm other studies (San-Miguel et al., 2006; Hu et al., 2020). Hu et al. (2020) showed that both the mRNA and protein levels of Nrf-2 were significantly reduced after RHDV infection, which shows that RHDV infection inhibits Nrf-2 activity and the antioxidant response. San-Miguel et al. (2006) showed that oxidative stress is a primary pathway for apoptosis in RHDV.

A detailed analysis of the first *HO-I* deficiency in a human showed that *HO-I* protects many tissues and organs against oxidative stress and excessive inflammatory responses by releasing many molecules with stress and antioxidant properties (Yachie, 2021). In addition, it protects against programmed cell death, and this cytoprotective effect is based on its ability to catabolize free heme and prevent cells from sensitizing to apoptosis. *HO-I* production is induced *in vivo* in selected cell types, including renal tubular epithelium, liver Kupffer cells, vascular endothelium, and monocytes/macrophages, suggesting that *HO-I* plays a crucial role in these cells. Data from reported cases of *HO-I* deficiency in humans and numerous studies in animal models suggest that *HO-I* plays a crucial role in various clinical conditions involving oxidative stress (Yachie, 2021). The magnitude of HO-I induction after oxidative stress and the wide distribution of this enzyme in systemic tissues, combined with the biological activity of the catalytic byproducts carbon monoxide, iron, and bilirubin, make HO-I a very attractive and interesting biomarker of oxidative stress and may play an essential role in mediating protection against liver, lung, spleen, and kidney damage (Choi and Alam, 1996). Because of the above facts, our results indicate that the reduction of *HO-I* mRNA at the level of 52%–69% in rabbit organs (liver, kidneys, spleen) after infection with *L. europaeus* GI.1 and GI.2 genotypes does not protect cells against oxidative damage but, on the contrary, may intensify, which is consistent with the results of experimental studies on various animal models (Choi and Alam, 1996; Yachie, 2021). The increase in *HO-I* mRNA expression (2-fold change, *p* = 0.02 vs. control) in the lungs during *L. europaeus* GI.2 infection, observed in our studies, indicates a role in protection against lung damage. Accumulating evidence suggests that oxidative stress plays a central role in the pathogenesis of many pulmonary diseases, including adult respiratory distress syndrome, emphysema, asthma, bronchopulmonary dysplasia, and interstitial pulmonary fibrosis (Choi and Alam, 1996).

### 4.1 Proposed miRs, target genes, and pathways apoptosis and oxidative stress during *Lagovirus europaeus* GI.1 and GI.2 infection

**Figure 8:**
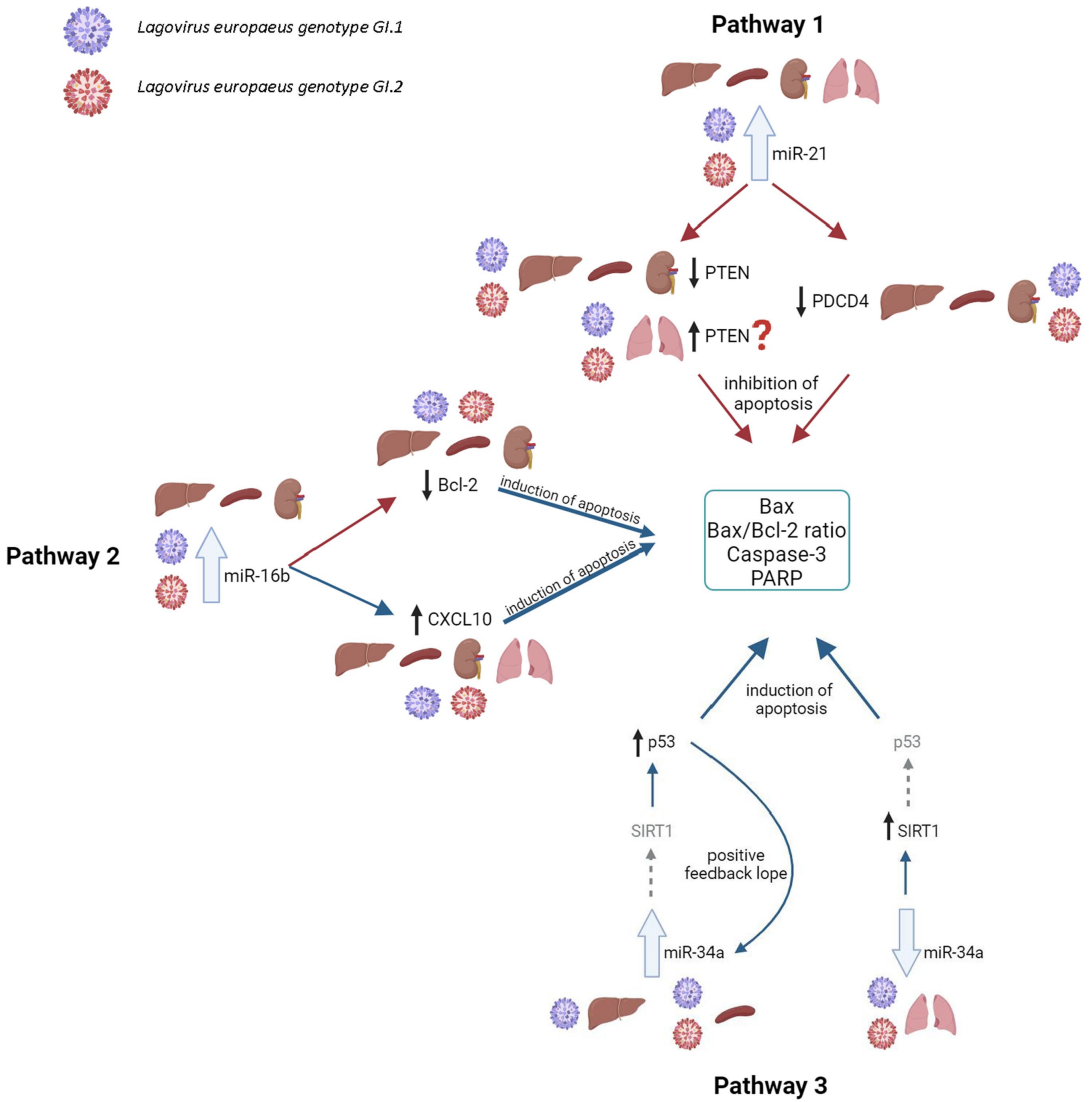
Contribution of miRs/target genes involved in the apoptosis pathway in *Lagovirus europaeus* infection. **Pathway 1**—increase miR-21 expression during *L. europaeus—*GI.1 and GI.2 genotypes inhibit *PTEN* and *PDCD4* in the liver (*PTEN*—Spearman’s rank correlation Rho: −0.74, *p* = 0.013, *PDCD4*—Spearman’s rank correlation Rho: −0.73, *p* = 0.015 for GI.1 and *PTEN*—Spearman’s rank correlation Rho: −0.68, *p* = 0.029, *PDCD4*—Spearman’s rank correlation Rho: −0.78, *p* = 0.007 for GI.2), kidney (*PTEN*—Spearman’s rank correlation Rho: −0.84, *p* = 0.002, PDCD4—Spearman’s rank correlation Rho: −0.83, *p* = 0.003 for GI.1 and *PTEN*—Spearman’s rank correlation Rho: −0.66, *p* = 0.038, *PDCD4*—Spearman’s rank correlation Rho: −0.72, *p* = 0.019 for GI.2), and spleen (*PTEN*—Spearman’s rank correlation Rho: −0.64, *p* = 0.048, *PDCD4*—Spearman’s rank correlation Rho: −0.71, *p* = 0.022 for GI.1 and *PTEN*—Spearman’s rank correlation Rho: −0.78, *p* = 0.008, *PDCD4*—Spearman’s rank correlation Rho: −0.94, *p* < 0.001 for GI.2) and effect on *Caspase-3* (in kidney Spearman’s rank correlation Rho: 0.7, *p* = 0.025 for GI.1, in spleen Spearman’s rank correlation Rho: 0.84, *p* = 0.002 for GI.1 and Spearman’s rank correlation Rho: 0.89, *p* = 0.0005 for GI.2), *Bax* (Spearman’s rank correlation Rho: 0.66, *p* = 0.04) and *Bax/Bcl-2* ratio (Spearman’s rank correlation Rho: 0.68, *p* = 0.029) in the liver for GI.1. The exception is the lungs, where no inhibitory mechanism is observed during infection with *L. europaeus* in both genotypes. **Pathway 2**—increase miR-16b expression during *L. europaeus* GI.1 and GI.2 in the liver (Spearman’s rank correlation Rho: −0.64, *p* = 0.043 for GI.1 and Spearman’s rank correlation Rho: −0.84, *p* = 0.002 for GI.2), spleen (Spearman’s rank correlation Rho: −0.85, *p* = 0.002 for GI.1 and Spearman’s rank correlation Rho: −0.74, *p* = 0.013 for GI.2), and kidney (Spearman’s rank correlation Rho: −0.77, *p* = 0.009 for GI.1 and Spearman’s rank correlation Rho: −0.64, *p* = 0.043 for GI.2) inhibit the *Bcl-2* target gene, which is an antiapoptotic gene. In this case, the exception is the lungs, where after infection with both *L. europaeus* genotypes, we do not observe a change in the expression of miR-16b and *Bcl-2* but an increase in the expression of the proapoptotic *Bax*. Moreover, miR-16b affects the growth of the chemoattractant *CXCL10* in the liver (Spearman’s rank correlation Rho: 0.77, *p* = 0.009 for GI.1 and Spearman’s rank correlation Rho: 0.79, *p* = 0.006 for GI.2), spleen (Spearman’s rank correlation Rho: 0.73, *p* = 0.016 for GI.1 and Spearman’s rank correlation Rho: 0.75, *p* = 0.013 for GI.2), and kidney (Spearman’s rank correlation Rho: 0.63, *p* = 0.048 for GI.1 and Spearman’s rank correlation Rho: 0.76, *p* = 0.011 for GI.2). The exception is the lungs, where no change in miR-16b expression was observed, but an increase in *CXCL10* expression was observed in both genotypes. Additionally, in the liver, miR-16 correlates with the *Bax/Bcl-2* ratio (Spearman’s rank correlation Rho: 0.63, *p* = 0.047 for GI.1) and with *Caspase-3* (Spearman’s rank correlation Rho: 0.83, *p* = 0.003 for GI.2). In the kidney, the analysis showed a positive correlation of miR-16 with the *Bax/Bcl-2* ratio (Spearman’s rank correlation Rho: 0.85, *p* = 0.002 for GI.1 and Spearman’s rank correlation Rho: 0.89, *p* = 0.0005 for GI.2). Whereas correlation miR-16 with *Caspase-3* has been demonstrated only during infection of GI.2 in the kidney (Spearman’s rank correlation Rho: 0.67, *p* = 0.03) and in the spleen (Spearman’s rank correlation Rho: 0.71, *p* = 0.02). **Pathway 3**—increased miR-34a expression during *L. europaeus* GI.1 infection in the liver and during GI.1 and GI.2 in the spleen do not affect *SIRT1* expression but induce *p53* in the liver (Spearman’s rank correlation Rho: 0.68, *p* = 0.028 for GI.1) and spleen (Spearman’s rank correlation Rho: 0.73, *p* = 0.016 for GI.1 and Spearman’s rank correlation Rho: 0.77, *p* = 0.009 for GI.2; GI.1 and GI.2), which leads to increased apoptosis. Despite the lack of statistical significance in the increase in miR-34a in the liver during GI.2 infection, it correlates with a decrease (also statistically insignificant) in *SIRT1* mRNA level (Spearman’s rank correlation Rho: −0.75, *p* = 0.011). Additionally, *p53* positively correlates with biomarkers of apoptosis. In the liver, *p53* correlates with *Caspase-3* (Spearman’s rank correlation Rho: 0.65, *p* = 0.042 for GI.1 and Spearman’s rank correlation Rho: 0.63, *p* = 0.047 for GI.2) and with the *Bax/Bcl-2* ratio (Spearman’s rank correlation Rho: 0.75, *p* = 0.01 for GI.1 and Spearman’s rank correlation Rho: 0.77, *p* = 0.009 for GI.2). Whereas *p53* in the liver correlates with *Bax* only during GI.1 infection (Spearman’s rank correlation Rho: 0.8, *p* = 0.004). In the spleen, *p53* correlates with *Bax/Bcl-2* ration in both genotypes (Spearman’s rank correlation Rho: 0.78, *p* = 0.008 for GI.1 and Spearman’s rank correlation Rho: 0.81, *p* = 0.004 for GI.2) and with *Caspase-3* only in GI.2 (Spearman’s rank correlation Rho: 0.75, *p* = 0.01). A noticeable regulatory effect of miR-34a was observed in the lungs, which correlated with an increase in *SIRT1* (Spearman’s rank correlation Rho:−0.64, *p* = 0.042 for GI.1 and Spearman’s rank correlation Rho:−0.77, *p* = 0.009 for GI.2) did not affect the regulation of p53. ?—further research is necessary; the gray arrow indicates the lack of regulatory influence in our research.

**Figure 9:**
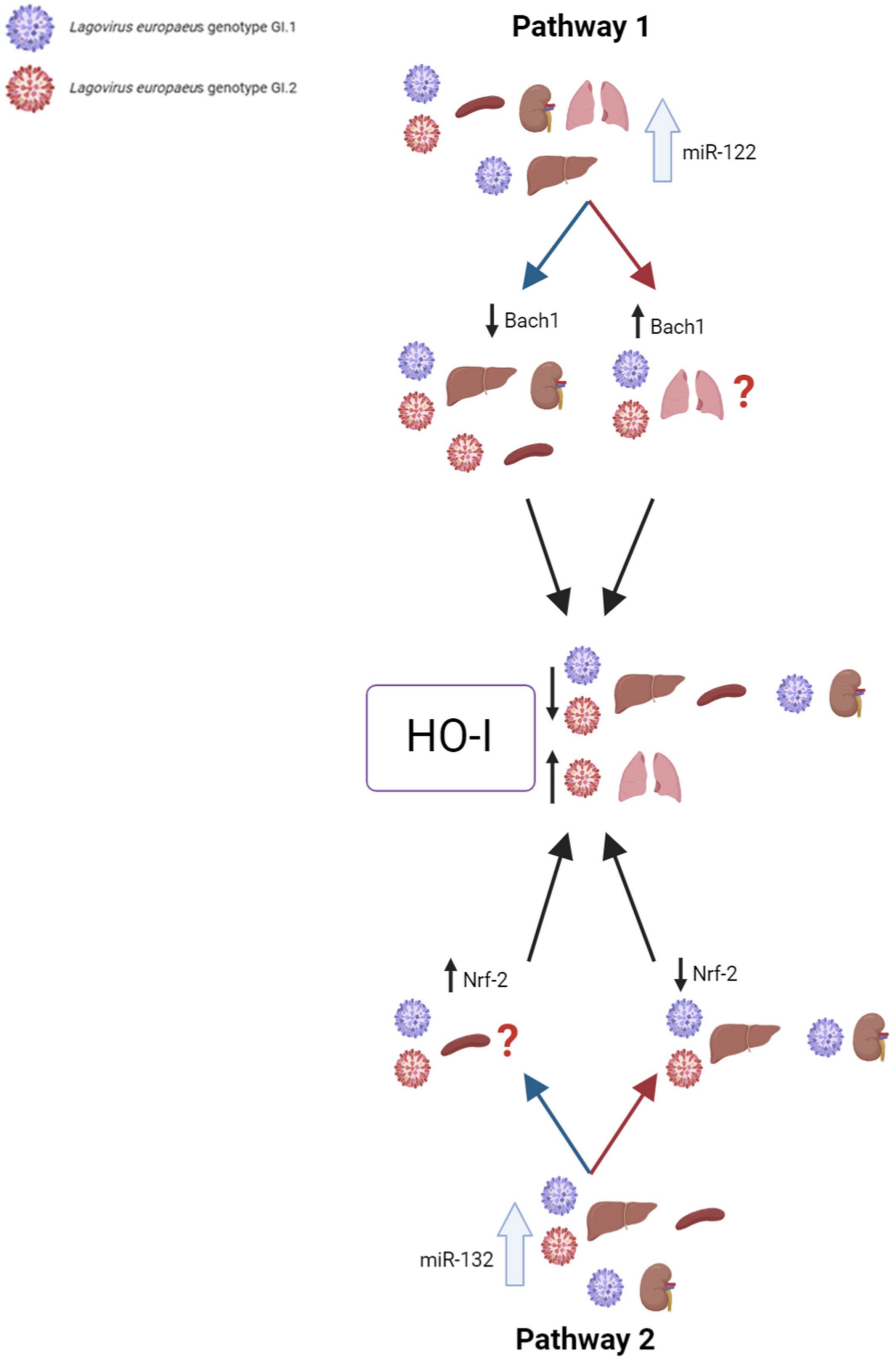
Contribution of miRs/target genes involved in the oxidative stress pathway in *Lagovirus europaeus* infection. **Pathway 1**—increase miR-122 expression during *L. europaeus* GI.1 and GI.2 genotype infection leads to a decrease in *Bach1* in all organs except in the lungs. miR-122 correlates with *Bach1* in the liver (Spearman’s rank correlation Rho: −0.71, *p* = 0.021 for GI.1 and Spearman’s rank correlation Rho: −0.66, *p* = 0.037 for GI.2), kidneys (Spearman’s rank correlation Rho: −0.76, *p* = 0.011 for GI.1 and Spearman’s rank correlation Rho: −0.8, *p* = 0.005 for GI.2), and spleen (Spearman’s rank correlation Rho: −0.85, *p* = 0.002 for GI.2). The upregulation of miR-122 leads to a decrease in *HO-I* mRNA levels in the liver (Spearman’s rank correlation Rho: −0.95, *p* < 0.001 for GI.1 and Spearman’s rank correlation Rho: −0.7, *p* = 0.025 for GI.2), spleen (Spearman’s rank correlation Rho: −0.81, *p* = 0.004 for GI.1 and Spearman’s rank correlation Rho: −0.92, *p* = 0.0001 for GI.2), and kidney (Spearman’s rank correlation Rho: −0.91, *p* = 0.0002 for GI.1 and Spearman’s rank correlation Rho: −0.79, *p* = 0.006 for GI.2), which leads to increased tissue damage. Therefore, *HO-I* has no protective effect on *L. europaeus* infection (except for the lungs). **Pathway 2**—it has not been previously described in viral infections, so it is a novelty. miR-132 expression during *L. europaeus* GI.1 and GI.2 in the liver, spleen, and kidney (only GI.1) inhibits *Nrf-2* in the liver (Spearman’s rank correlation Rho: −0.83, p = 0.003 for GI.1 and Spearman’s rank correlation Rho: −0.7, *p* = 0.025 for GI.2) and kidneys (Spearman’s rank correlation Rho: −0.87, *p* = 0.001 for GI.1). Further research is needed to identify the factor influencing the increase in *Nrf-2* expression in the spleen during infection with *L. europaeus*. The correlation analyses performed indicate miR-122 as the main inhibitor of *HO-I* levels (not *Bach1* or *Nrf-2*) during *L. europaeus* infection, which can lead to increased tissue damage. ?—further research is necessary.

Our research proposes five pathways in *L. europaeus* infection, three of which are involved in apoptosis pathways and two in oxidative stress (Figures 8, 9). Additionally, Spearman’s rank correlations for examined miRs, mRNA, and biomarkers of processes in four tissues of rabbits during *L. europaeus* GI.1 and GI.2 infection were described. Correlations are provided for statistically significant results (Figures 8, 9).

## 5 Conclusion

Our report is the first to present the regulatory effects of miRs on apoptosis and oxidative stress genes in rabbit infection with *L. europaeus*—two genotypes (GI.1 and GI.2) in four tissues (liver, lungs, kidneys, and spleen). Our research provides new data that are critical for understanding the pathogenesis of Rabbit Hemorrhagic Disease caused by *L. europaeus*—two genotypes (GI.1 and GI.2), regarding the molecular regulation of apoptosis and oxidative stress by miRs (as two essential biological processes in viral infections). The regulatory effect of miRs indicates that, on the one hand, miRs can intensify apoptosis (miR-16b, miR-34a) in the examined organs in response to a viral stimulus and, on the other hand, inhibit (miR-21), which in both cases may be a determinant of the pathogenesis of RHD and tissue damage. Biomarkers of the *Bax and Bax/Bcl-2* ratio promote more intense apoptosis after infection with the *L. europaeus* GI.2 genotype. Our findings demonstrate that miR-122 and miR-132 regulate oxidative stress in the pathogenesis of RHD, which is associated with tissue damage. The *HO-I* biomarker in the course of rabbit hemorrhagic disease indicates oxidative tissue damage. Our findings show that miR-21, miR-16b, and miR-34a regulate three apoptosis pathways. Meanwhile, miR-122 and miR-132 are involved in two oxidative stress pathways. The results of our research also have diagnostic (searching for potential disease biomarkers) and therapeutic (modulating miR-dependent pathways) potential during acute liver failure (ALF) and multi-organ failure (MOF) of viral etiology, which we encounter during *L. europaeus* infection.

## Data availability statement

The original contributions presented in the study are included in the article/supplementary material, further inquiries can be directed to the corresponding author.

## Ethics statement

The animal study was approved by the Local Ethical Committee for Animal Experiments in Poznań, Poland (no. 51/2022). The study was conducted in accordance with the local legislation and institutional requirements.

## Author contributions

EO: conceptualization, data curation, investigation, methodology, software, validation, visualization, writing—original draft, and writing—review and editing. AF: investigation, resources, and writing—original draft. AK: investigation, resources, and writing—original draft. AS: investigation, resources, and writing—original draft. BHS: conceptualization, data curation, formal analysis, investigation, methodology, supervision, validation, writing—original draft, writing—review and editing, funding acquisition, and project administration.
